# 1040. Knee Explant Analysis (KnEA) Using PLG0206 in Periprosthetic Joint Infection (KnEA Study)

**DOI:** 10.1093/ofid/ofab466.1234

**Published:** 2021-12-04

**Authors:** David Huang, Dana Parker, Nicholas Pachuda, Despina Dobbins, Jonathan Steckbeck, Kenneth Urish

**Affiliations:** 1 Peptilogics, Houston, Texas; 2 University of Pittsburgh, Pittsburgh, Pennsylvania

## Abstract

**Background:**

PLG0206 is a novel engineered cationic antimicrobial peptide being evaluated for treatment of prosthetic joint infections (PJI). This study evaluated the rapid bactericidal activity of PLG0206 to decrease biofilm and planktonic bacteria on *ex vivo* infected prosthesis following removal from patients with chronic PJI.

**Methods:**

De-identified infected prosthetics were removed from nine patients with PJI, despite chronic suppressive oral antibiotics, during a 2-stage revision procedure. Removed prosthetics were then submersed *ex vivo* to an expected clinical exposure of PLG0206, 1 mg/mL, for ~15 minutes. Upon completion of the 15-minute exposure, the treated explant was placed into buffer and sonicated. The sonication solution was then plated for bacterial analysis including colony forming unit (CFU) enumeration. Remaining explanted implants from the same patient served as a control and was processed similarly but without exposure to PLG0206.

**Results:**

As shown in the Table, both Gram-positive and Gram-negative bacteria were identified from removed prosthetics during a 2-stage revision procedure of chronic PJI. Eight of ten infected prosthetics treated *ex vivo* to PLG0206 1 mg/mL were sterilized (No. 1-5, 8-10). Of the two infected prosthetics that were not sterilized (No. 6 and 7), one was polymicrobial (No. 6) and the other was monomicrobial (No. 7). Collectively, infected prosthetics exposed to PLG0206 demonstrated a mean 4log10 reduction (range 2 to 7).

Summary of culture and CFU log reduction among infected prosthetics exposed and not exposed to PLG0206

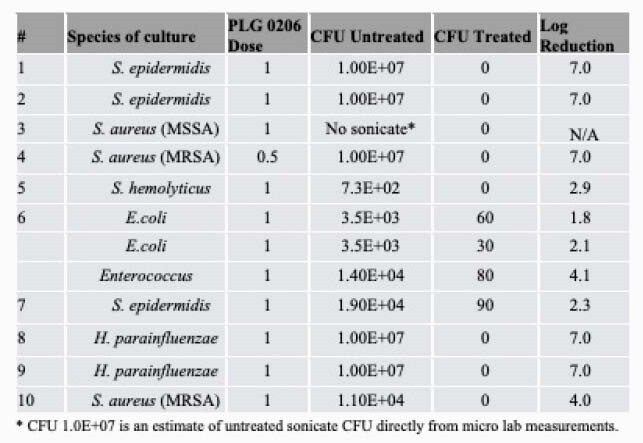

Table: Summary of culture and CFU log reduction among infected prosthetics exposed and not exposed to PLG0206

**Conclusion:**

Overall, these findings support the ongoing development of PLG0206 as a local irrigation solution at 1 mg/mL concentration in the wound cavity for 15 minutes in patients undergoing treatment of a PJI occurring after total knee arthroplasty.

**Disclosures:**

**David Huang, MD, PhD**, **Peptilogics** (Employee) **Nicholas Pachuda, DPM**, **Peptilogics** (Employee) **Despina Dobbins, BS**, **Peptilogics** (Employee) **Jonathan Steckbeck, PhD**, **Peptilogics** (Employee) **Kenneth Urish, MD, PhD**, **Peptilogics** (Grant/Research Support)

